# Amplitude Dependence of Resonance Frequency and its Consequences for Scanning Probe Microscopy

**DOI:** 10.3390/s19204510

**Published:** 2019-10-17

**Authors:** Omur E. Dagdeviren, Yoichi Miyahara, Aaron Mascaro, Tyler Enright, Peter Grütter

**Affiliations:** Department of Physics, McGill University, Montreal, QC H3A 2T8, Canada; yoichi.miyahara@mcgill.ca (Y.M.); mascaroa@physics.mcgill.ca (A.M.); tyler.enright@mcgill.mail.ca (T.E.); peter.grutter@mcgill.ca (P.G.)

**Keywords:** resonance frequency, amplitude dependence of resonance frequency, scanning probe microscopy, atomic force microscopy, frequency modulation atomic force microscopy, noncontact atomic force microscopy

## Abstract

With recent advances in scanning probe microscopy (SPM), it is now routine to determine the atomic structure of surfaces and molecules while quantifying the local tip-sample interaction potentials. Such quantitative experiments using noncontact frequency modulation atomic force microscopy is based on the accurate measurement of the resonance frequency shift due to the tip-sample interaction. Here, we experimentally show that the resonance frequency of oscillating probes used for SPM experiments change systematically as a function of oscillation amplitude under typical operating conditions. This change in resonance frequency is not due to tip-sample interactions, but rather due to the cantilever strain or geometric effects and thus the resonance frequency is a function of the oscillation amplitude. Our numerical calculations demonstrate that the amplitude dependence of the resonance frequency is an additional yet overlooked systematic error source that can result in nonnegligible errors in measured interaction potentials and forces. Our experimental results and complementary numerical calculations reveal that the frequency shift due to this amplitude dependence needs to be corrected even for experiments with active oscillation amplitude control to be able to quantify the tip-sample interaction potentials and forces with milli-electron volt and pico-Newton resolutions.

## 1. Introduction

Resonant structures are widely used as accurate measurement devices in fields of science ranging from biological chemical detection to gravitational waves or quantum mechanical systems [1,2,3,4,5]. Additionally, oscillating structures play a transducer role in atomic force microscopy (AFM) and related techniques [6,7]. Dynamic AFM is an analytical surface characterization tool where a sharp probe tip is mounted to the end of an oscillating probe which serves as a frequency sensing element to measure surface properties [6,7,8]. Among different dynamic AFM methodologies [6,7,9], the frequency modulation AFM (FM-AFM) technique is the dominant high-resolution material characterization method under ultra-high vacuum conditions [7,8,10]. The FM-AFM technique tracks the change in the resonance frequency of the cantilever, Δ*f*, under the influence of the attractive (or repulsive) surface forces while keeping the oscillation amplitude ‘*constant*’ [8]. Even though the physical foundations of variations of the device resonance frequency was a long-lasting problem that was postulated in mid-1970s [11,12], only recently it has been systematically demonstrated that the geometric and stress-induced deviations can modify the resonance frequency of micro-cantilever beams [13,14,15,16]. Here, we explore the amplitude dependence of the resonance frequency for the most commonly used oscillating probes in dynamic SPM measurements, as follows: Tuning forks, cantilever beams, and tuning forks in the qPlus configuration (which have one free prong to which the tip is attached to the end while the fork’s other prong is fixed to a holder [17], see Figure S1 and part I of the Supplemental Materials for further details.

Our experimental results reveal that the resonance frequency of oscillating probes changes by a nonnegligible amount within practical operating conditions. The resonance frequency of tuning forks and tuning forks in the qPlus configuration decreases with increasing oscillation amplitude. The decrease in the resonance frequency implies that the amplitude dependence of the resonance frequency of tuning forks and tuning forks in the qPlus configuration is dominated by in-plane stress near the clamped end of the beam, i.e., surface stress effect [13,14,15,16]. In contrast, the resonance frequency of cantilever beams increases with the oscillation amplitude due to geometric effects, i.e., geometry change due to elastic deformation with the application of a load [14,15]. With recent advances in scanning probe microscopy methods, atomically-engineered chemically identified tips can be obtained by attaching a molecule or an inert atom to the end of a scanned probe [18,19]. Chemically identified tips enable measurement of the local tip sample interaction deep into the repulsive regime [18,19,20]. Quantitative potential energy and force measurements with milli-electron volt and pico-Newton resolutions have recently been reported (*for some cases higher potential energy accuracies are proposed*). The currently reported experimental work can be summarized as the quantitative measurement of internal structures of molecules and chemical bonds in a molecule [21], the quantitative assessment of intermolecular interactions [22], the quantification of stiffness and the interaction with lateral force microscopy of molecules with sub milli-electron volt resolution for potential energy and zepto-Newton resolution for the torsional stiffness [23], and van der Waals interactions of isolated atoms with sub-milli-electron volt potential energy resolutions [24]. In parallel to these selected examples, investigating the validity and the accuracy of methods to extract the potential energy (and/or force) spectroscopy from experimental data and associated mathematical methods and approximations are ongoing research efforts [25,26]. Many experiments involve measuring the resonance frequency as a function of tip-sample separation in order to quantify the interaction potential as a function of distance, often with lateral atomic resolution [18,19,21,22,23,24,27,28,29]. These frequency measurements allow the experimental determination of surface chemical or electronic properties, with sub-nanometer spatial resolution.

We conducted numerical calculations to explore consequences of the amplitude dependence of the resonance frequency for high-resolution AFM measurements. In determining interaction forces and potentials from frequency shift data, it is assumed that the resonance frequency is independent of oscillation amplitude. In the following, we investigate the validity of this assumption and demonstrate that the systematic errors introduced by this assumption are comparable (or, in some cases even larger) than the precision of interaction potentials reconstructions reported in the literature. Our experiments and numerical calculations address the amplitude dependence of the resonance frequency as an important yet overlooked systematic error source which can impede the accurate measurement of the tip-sample interaction potential and force with milli-electron volt and pico-Newton resolutions. Therefore, the systematic error due to the amplitude dependence of the resonance frequency should be corrected for meaningful and accurate data acquisition.

## 2. Results

**Figure 1:**
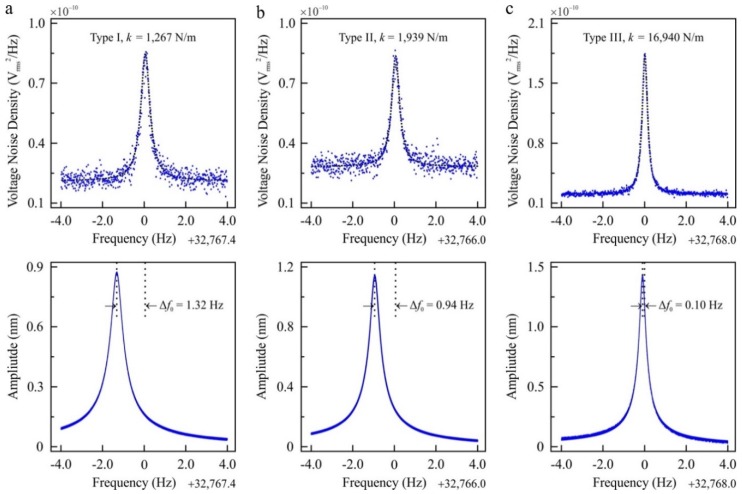
The measurement of the resonance frequency of encapsulated tuning forks with thermal noise density (first row, **a**–**c**) and frequency sweep experiments (second row, **a**–**c**). A Lorentzian curve is fitted to the thermal noise density and the resonance frequency is calculated from the fit. Thermal noise spectra presented in this figure were recorded at room temperature and averaged 100 times. The oscillation amplitude of tuning forks is calibrated with the principle of energy dissipation [30]. The frequency resolution of the thermal noise spectra is 4 milli-Hz while the frequency resolution of the resonance sweep experiments for encapsulated tuning forks are 0.4 milli-Hz (see Supplemental Materials part II for further details of experimental techniques). The experimental data presented in this figure was conducted with three different types of quartz tuning forks. While the tuning forks have the same resonance frequency (~2^15^ Hz), effective spring constants differs due to the difference in their geometric dimensions (see Table S1 for details).

We experimentally investigated the variation of the resonance frequency of tuning forks, tuning forks in the qPlus configuration, and cantilevers as a function of oscillation amplitude (see Supplemental Materials part II for details of experimental methods). We conducted experiments with three different types of quartz tuning forks. The tuning forks have the same resonance frequency (~2^15^ Hz), while their effective spring constant changes due to their geometric dimensions (see Table S1 for details). Figure 1 shows, the resonance frequency of different types of encapsulated tuning forks were measured with thermal noise spectra and frequency sweep experiments (see Figures S2–S4 for additional experimental results). Oscillation amplitudes of tuning forks are calibrated with the principle of energy dissipation, details of which can be found elsewhere [30]. As highlighted in Figure 1, the resonance frequency of excited tuning forks decreases with respect to the thermal excitation measurements. The viscous effects of the surrounding medium can be excluded as the tuning forks are encapsulated (i.e., in vacuum) [17]. In addition, piezoelectric nonlinearities can be excluded as current-induced piezoelectric nonlinearities increase the resonance frequency of *z*-cut piezoelectric devices rather than decreasing the resonance frequency, as we observed in our experiments [31,32]. Both thermal noise density and frequency sweep experiments are conducted successively in a temperature controlled and quiet room in a thermally isolated chamber. With the other effects eliminated, the drop of the resonance frequency implies that the effect of in-plane surface stress is the governing factor for the amplitude dependence of the resonance frequency [13,14,15,16]. The first mode of tuning forks is along the vertical direction with respect to the sample surface, which induces an in-plane stress (i.e., the stress is in the same plane as the mechanical oscillation) near the clamp.

In-plane stress results in a drop of the resonance frequency and the relative frequency shift due to surface stress, as demonstrated experimentally and theoretically in References [13,14,15,16], as follows:

(1) $$\frac{\Delta w}{w_{0}}=-0.042\frac{v\left(1-v\right)\sigma_{s}^{T}}{k_{eff}}$$

In Equation (1), Δw is the relative frequency shift, $w_{0}$ is the resonant frequency in the absence of the surface stress, v is the Poisson’s ratio, $\sigma_{s}^{T}$ is the total surface stress, and $k_{eff}$ is the stiffness of the cantilever beam in the absence of the surface stress. According to Equation (1), a frequency shift due to the surface stress is a function of the material of the oscillating beam (due to Poisson’s ratio), $\sigma_{s}^{T}$, and inversely proportional to $k_{eff}$. Additionally, Equation (1) implies that the product of Δw with $k_{eff}$ remains constant for the same range of total surface stress. As expected, based on this model, the product of the relative frequency shift and the effective spring constant of the three different size quartz tuning forks characterized here is 3460 ± 160 (kg/s^3^) (*k_eff_* = 2*k*, where *k* is the stiffness of a single prong, also see Supplemental Materials part IV). This supports our interpretation that the surface stress effect (Equation (1)) describes the amplitude dependence of the resonance frequency (also see Supplemental Materials part III and part IV for further discussion).

**Figure 2:**
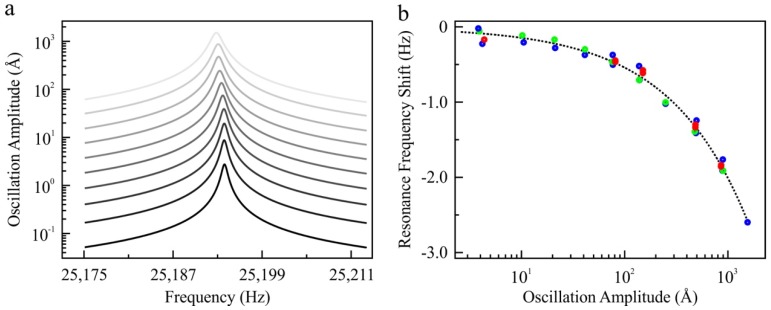
The measurement of the resonance frequency of a tuning fork (type-II, see Supplemental Materials part II for details of the tuning fork used for this specific experiment) in the qPlus configuration. Frequency sweep experiments (**a**) were conducted to determine the resonance frequency. The oscillation amplitude of the qPlus is calibrated with the principle of energy dissipation, which has a picometer range accuracy [30]. The frequency resolution of resonance sweep experiments is 10 milli-Hz for tuning forks in the qPlus configuration (see Supplemental Materials part II for further details of experimental techniques). (**b**) We fit a curve to experimental data (black, dashed line, *R*^2^ = 0.99 for the fit), $\Delta f=-0.03548\times A^{0.584}$, where Δf is the resonance frequency shift in Hz and *A* is the oscillation amplitude in Ångströms. To eliminate the possibility that this observation was due to Duffing nonlinearity and other sources of nonlinearities (e.g., temperature change due to energy dissipation during oscillation), we conducted successive experiments under different excitation conditions. We increased the resonance frequency during the sweep experiments and the oscillation amplitude for successive sweeps (blue circles). In addition, we decreased the frequency during resonance sweep experiments (red circles) for different oscillation amplitude in a consecutive experiment (green circles). To eliminate the effect of viscous damping and temperature variations, experiments presented in this figure were conducted at liquid nitrogen temperature (~77 K) in vacuum conditions (2 × 10^−7^ mbar).

Quartz tuning forks that have one free prong, to which the tip is attached to the end while the fork’s other prong is fixed to a holder (‘qPlus’ configuration), have gained popularity in recent years for high-resolution imaging (see Supplemental Materials part I for details) [17]. Figure 2 shows the decrease in resonance frequency with increasing oscillation amplitude of a qPlus sensor. We conducted successive frequency sweep experiments in which we increased and decreased the frequency to investigate the potential contribution of Duffing nonlinearity to the change in resonance frequency [33]. The identical same resonance curves were found independent of the oscillation amplitudes tested, establishing our system as a harmonic oscillator. The results in Figure 2 (also Figures S2–S4 for additional experimental data) show that the resonance frequency is a function of amplitude even in the small oscillation amplitude range (Ångströms to nanometers). The variation of the resonance frequency is more emphasized for tuning forks in the qPlus configuration compared to encapsulated tuning forks (see Figures S2 and S3). The increased effect of the oscillation amplitude on the resonance frequency can be linked to the sensor assembly, which can alter the stress concentration near the clamp [13].

For completeness, we also performed measurements of the amplitude dependence of the resonance frequency with conventional micro-fabricated silicon cantilevers, which are widely used for scanning probe microscopy experiments [6,7]. We compared thermal noise spectrum and frequency sweep experiments for two different types of cantilevers (cantilever-I OPUS 4XC-NN-A, cantilever-II OPUS 4XC-NN-B) in vacuum. As Table 1 (also Figure S5 for additional experimental data) summarizes, the resonance frequency of cantilevers increases with increasing oscillation amplitude in contrast to encapsulated tuning forks and tuning forks in the qPlus configuration, where the resonance frequency decreases with increasing amplitude. Our experiments with cantilever beams are in line with former experimental and theoretical work which predicts that the resonance frequency of cantilever beams increases due to geometric effects [14,15].

## 3. Discussion

In the following, we investigate what the consequences are in the field of non-contact AFM (NC-AFM) for the amplitude dependent resonant frequency. In NC-AFM, measurements of the resonance frequency as a function of tip-sample separation are often used to reconstruct the sample’s potential energy landscape. This is known potential energy (and/or force) spectroscopy and used to understand reaction pathways, diffusion, as well as to validate ab-initio modeling. To demonstrate the effect of the amplitude dependent frequency, we conducted numerical calculations to determine the consequences on the potential energy and force spectroscopy experiments. Even though the amplitude is intended to be ‘*kept constant*’ via a feedback mechanism during the frequency modulation-based force spectroscopy experiments, a non-zero amplitude error is always present [8]. To understand the effect of the amplitude error, we fitted an empirical curve to the experimental data presented in Figure 2b (see figure caption and Supplemental Materials for details). We used Equation (2) to calculate the resonance frequency shift due to the amplitude error around the amplitude setpoint, as follows:

(2) $$\Delta f_{error}=\Delta f_{fit}\left(A_{set}\right)-\Delta f_{fit}\left(A_{set}+A_{error}\right)$$

where $\Delta f_{error}$ is the frequency shift due to amplitude dependence of resonance frequency, $A_{set}$ is the amplitude setpoint, and $A_{error}$ is the amplitude error using the fitted curve ($\Delta f_{fit}$) to the experimental data in Figure 2b. In experiments, a non-zero amplitude error is present due to the limited bandwidth and the accuracy of measurement electronics. We assume an $A_{error}$ of 10 picometers to be a constant as a function of tip-sample distance. Note that in reality, however, $A_{error}$ can increase more significantly in the proximity of the surface and can have a larger error corrugation across the scan area due to non-linear tip-sample interaction and due to the effect of dissipative forces [9,35]. For this reason, the assumption of a constant $A_{error}$ underestimates the amplitude error with respect to experimental conditions where the effect of dissipative tip-sample interactions can further enhance the amplitude variations with decreasing tip-sample distance [35].

To demonstrate the consequences of the amplitude dependent resonance frequency and the pathway to correct this effect, we calculated the resonance frequency shift due to a tip-sample interaction potential as a function of $A_{set}$ and then reconstructed the tip-sample interaction potential using well-established mathematical procedures with and without considering the effect of $A_{error}$ on the frequency shift (see Supplemental Materials part V for details of numerical calculations) [36].

**Figure 3:**
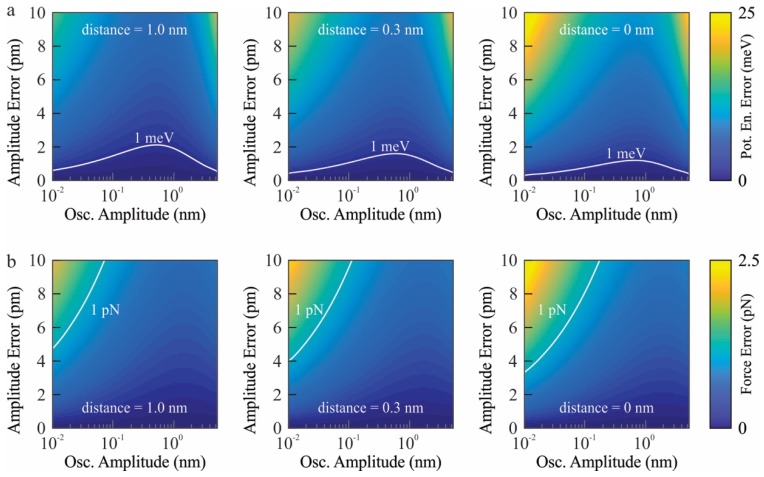
The effect of amplitude dependence of the resonance frequency on recovered tip-sample interaction potential (**a**) and force (**b**) of tip-sample distance and amplitude error. (**a**) The error of recovered tip-sample interaction potential increases with decreasing tip-sample separation. To keep the error less than one milli-electron volt and one pico-Newton (white contour in (**a**,**b**)), the amplitude error should be less than one picometer. If the amplitude error is not constraint to sub-picometer range, the frequency shift due to the amplitude dependence of the resonance frequency should be corrected. By this way, the intrinsic contribution of frequency shift due to tip-sample interaction can be calculated and error in the reconstructed tip-sample interaction potential and force can be eliminated. The reference point for the distance, i.e., distance = 0, is defined as the potential energy minimum of the model tip-sample interaction (see Supplemental Materials for details).

As Figure 3 summarizes, we investigated the error in the reconstructed potential energy and force as a function of amplitude error ($A_{error}$), amplitude setpoint ($A_{set}$), and tip-sample distance. As Figure 3a,b reveals, unless the amplitude error is minimized or corrected, the amplitude dependence of the resonance frequency, $\Delta f_{error},$ does not allow measurements with milli-electron volt and pico-Newton accuracy. We note that the systematic error due to the amplitude dependence of the resonance frequency is a significant, yet overlooked, source of experimental error given the fact that the tip-sample interaction potential of high-resolution spectroscopy experiments is on the order of a few tens of milli-electron volts and force in the order of pico-Newtons [21,22,23,24]. The error induced by the amplitude dependence of the resonance frequency has two major trends depending on the oscillation amplitude. As highlighted by Figure 3, the error of the reconstructed tip-sample interaction potential and force peaks when $A_{set}$ is comparable to $A_{error}$. In addition to this trivial trend, the effect of amplitude error increases when $A_{set}$ is on the order of nanometers. This is due to the stronger dependence of the resonance frequency on amplitude for oscillation amplitudes larger than a few nanometers. As Figure 2 and Figures S2–S4 demonstrate, the amplitude-dependent change in resonance frequency, $\Delta f_{error}$, becomes more important with increasing oscillation amplitude.

Our results highlight that the amplitude dependence of the resonance frequency is a systematic error source due to the intrinsic properties of the oscillating probe used, independent of the nature of tip-sample interaction potential (see Supplemental Materials part V for details). For this reason, the error induced due to the amplitude dependence of the resonance frequency will persist even for a mathematically well-posed interaction [25,26]. As a consequence, most experimental force spectroscopy data derived from frequency shift measurements presented in the literature are expected to have systematic errors that can be corrected for. As Equation (3) summarizes, by measuring the oscillation amplitude ($A_{osc}$) dependence of the resonance frequency for the sensor in use, e.g., Figure 2b, and the oscillation amplitude error, the contribution of amplitude error on quantitative spectroscopy experiments can be eliminated (see Supplemental Materials part VI for the detailed algorithm).

(3) $$\Delta f_{tip-sample}=\Delta f_{measured}-\Delta f_{error}$$

Our numerical calculations show that unless the resonance frequency shift due to amplitude dependent resonance frequency error is corrected, the oscillation amplitude error has to be kept less than one picometer to achieve milli-electron volt and piconewton resolution. Controlling the oscillation amplitude with sub-picometer error corrugation across the scan area is not straightforward at all due to hardware related experimental limitations [7,9]. We want to emphasize that the error induced by the amplitude dependence of the resonance frequency is an intrinsically related to the oscillating probe characteristics (see Equation (1)) and is independent of the tip-sample interaction (see Supplemental Materials part V for details). Therefore, the effect of the amplitude dependence of the resonance frequency should be corrected with our proposed algorithm (see Supplemental Materials part VI) for the quantitative measurement of any tip-sample interaction potentials and forces with milli-electron volt and pico-Newton resolutions, respectively.

As discussed at the beginning of results and discussion section, current-induced piezoelectric nonlinearities of *z*-cut piezoelectric devices, temperature fluctuations, and acoustic noise may lead to a variation of the resonance frequency [31,32]. Current piezoelectric nonlinearities of *z*-cut piezoelectric devices increase the resonance frequency with increasing oscillation amplitude rather than the decrease, as we observed in our experiments for tuning forks. Additionally, we conducted our experiments in a temperature-controlled room in an acoustically isolated chamber. Even though other mechanisms are eliminated, we want to emphasize that any amplitude dependence of the resonance frequency needs to be corrected to avoid systematic errors in the reconstructed force or potential values. In addition, possible implications of the amplitude dependent resonance frequency are not limited to force spectroscopy experiments, for instance, multimodal atomic force microscopy, in which the relative amplitudes of different harmonics can potentially change due to the amplitude dependence of resonance frequency. With the change of the modal shape, the amplitude dependence of the resonance frequency of higher harmonics can be different. This may further complicate the data interpretation and the correction. Furthermore, the optical drive or the thermal drive of oscillating probes may enhance non-linearities. This can promote additional non-linearity mechanisms due to temperature fluctuations. Moreover, Kelvin probe force microscopy and pump-probe atomic force microscopy measurements require a precise measurement of the resonance frequency. For this reason, the amplitude dependence of the resonance frequency can challenge the accuracy of quantitative Kelvin probe force microscopy and pump-probe AFM measurements and time-resolved non-contact atomic force microscopy experiments [37,38]. We also want to mention that the existence of vacuum conditions is not a prerequisite for the amplitude dependence of the resonance frequency (see Table 1, also Figures S3–S5), which may alter experiments and data interpretation of infra-red AFM measurements under ambient conditions. Such experiments rely on the demodulation of the cantilever resonance in close proximity to an optically excited sample. With the change of the oscillation amplitude, the resonance frequency of different harmonics can be modified due to amplitude dependence of the resonance frequency. This may lead to resonance side bands or complications of the data analysis if a quantitative description is aimed for the light absorption of the illuminated tip-sample system.

## 4. Conclusions

We experimentally demonstrated that the resonance frequency of the most commonly used oscillating probes for dynamic atomic force microscopy experiments changes significantly as a function of oscillation amplitude. Amplitude errors need to be maintained below one picometer to keep resultant systematic energy and force errors below milli-electron volt and pico-Newton, respectively. Our results demonstrate that the amplitude dependence of the resonance frequency is a nonnegligible yet overlooked systematic error source for the quantitative measurement of tip-sample interaction potentials and forces if milli-electron volt and pico-Newton accuracy are claimed. Finally, we want to note that the amplitude dependence of the resonance frequency has possible consequences for other scanning probe microscopy methodologies.

## Figures and Tables

**Table 1:** The measurement of the resonance frequency of two different types of micro-cantilevers with thermal noise density and frequency sweep experiments. For both cantilevers, the resonance frequency increases with the oscillation amplitude. Experimental data are the average of 5 independent measurements of 50 spectra averaged together. The resonance frequency is determined by fitting a Lorentzian function to the thermal noise spectrum. The oscillation amplitude at the resonance frequency is 8.2 nm for cantilever-I and 7.5 nm for cantilever-II. The experimental setup was kept in a quiet room during the experiments to eliminate the effect of acoustic noise [34].

	f_0_ with Thermal Noise (Hz)	f_0_ with Frequency Sweep (Hz)
**Cantilever-I**	18156.8 ± 0.4	18163.4 ± 0.2
**Cantilever-II**	84325.9 ± 1.5	84329.0 ± 1.5

## Supplementary Materials

The following are available online at https://www.mdpi.com/1424-8220/19/20/4510/s1, Figure S1: Schematic representation of tuning forks in the qPlus configuration. Figure S2: The measurement of the resonance frequency of an encapsulated tuning fork as a function of oscillation amplitude. Figure S3: The measurement of the resonance frequency of a tuning fork in qPlus configuration. Figure S4: The measurement of the resonance frequency of a tuning fork in qPlus configuration. Figure S5: The measurement of the resonance frequency of a microfabricated silicon cantilever-I (OPUS 4XC-NN-A) as a function of oscillation amplitude. Figure S6: The assembled sensor setup and the modelling procedure to determine the modal shape and the surface stress distribution of tuning fork-based sensors. Figure S7: Calibration of spring constant with finite element methods. Figure S8: Summary of the algorithm to correct the resonance frequency shift data to eliminate the effect of the amplitude dependence of the resonance frequency. Table S1: Dimensions of tuning forks are measured with a calibrated light microscope. Table S2: Material properties used for finite element method (FEM) calculations.
