# Supplementary material for: Amplitude Dependence of Resonance Frequency and its Consequences for Scanning Probe Microscopy
Source: Sensors (Basel). 2019 Oct 17;19(20):4510. doi: 10.3390/s19204510 (PMC6832880; doi:10.3390/s19204510)
Supplement: Supplementary file 1 [file sensors-19-04510-s001.pdf]

sensors

Supplemental Materials

Amplitude Dependence of Resonance Frequency and its Consequences for Scanning Probe Microscopy

Omur E. Dagdeviren *, Yoichi Miyahara, Aaron Mascaro, Tyler Enright and Peter Grütter

Department of Physics, McGill University, Montréal, Québec, Canada, H3A 2T8
* Correspondence: omur.dagdeviren@mcgill.ca

I. Tuning Forks in the qPlus Configuration

Tuning forks are used commonly used as force sensing elements in different scanning probe microscopy techniques such as non-contact atomic force microscopy or scanning near-field optical microscopy [1,2]. Tuning fork-based sensors are popular due to the ability of the piezoelectric quartz to 'self-sense' deflections through the detection of induced surface charges [1,3]. In addition, it is possible to operate tuning forks with small oscillation amplitudes (Ångströms to nanometers) while avoiding mechanical instabilities with the high spring constant of tuning forks. Additionally, the flexibility in choice of the tip material allows to tailor the tip-sample interaction. Quartz tuning forks that have one free prong, to which the tip is attached to the end while the fork's other prong is fixed to a holder ('qPlus' configuration, see Figure S1), have gained popularity for high-resolution imaging in recent years [1]. The scanned probe tip is attached to the end of the oscillating prong and interacts with the surface. By decoupling the prongs of the tuning fork, data interpretation is simplified under the influence of tip-sample interactions [4].

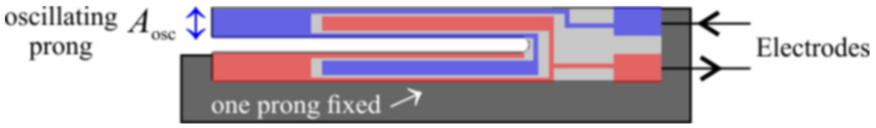

Figure S1: Schematic representation of tuning forks in the qPlus configuration. For tuning forks in the qPlus configuration, one of the prongs of the tuning fork is fixed while the other prong oscillates freely (also known as qPlus configuration). The mechanical oscillation, A_{osc}, induces a charge on the surface of the piezoelectric material. The induced charge is collected via electrodes and amplified. As the induced charge is correlated with the mechanical oscillation amplitude [4], the requirement of an optical detection mechanism is eliminated.

II. Experimental Methods and Additional Experimental Results

We conducted thermal noise spectra experiments and frequency sweep experiments to measure the resonance frequency of probes. The thermal noise spectra were obtained by fast Fourier transform of the detection signal. The thermal spectra tuning forks were obtained at room temperature by employing a low-noise preamplifier (Stanford Research Systems, Model SR560) operated at the differential input mode followed by fast Fourier transform of the detection signal. The driven frequency spectra were measured by a lock-in amplifier with a sinusoidal drive signal with constant amplitude while its frequency was swept. The frequency resolution of the thermal noise spectra is 4 milli-Hz. The frequency resolution of the driven frequency spectra is 0.4 milli-Hz for encapsulated tuning forks, 10 milli-Hz for tuning forks in the qPlus configuration, and 0.1 Hz for cantilevers. The resonance frequency was determined by fitting a Lorentzian curve to the experimental frequency spectra around the peak. The oscillation amplitude of tuning forks and tuning forks in the qPlus configuration are measured with the principle of energy dissipation with picometer range accuracy, details of which can be found elsewhere [4]. All experiments were conducted at room temperature

(unless otherwise stated) in a quiet room to avoid the contribution of acoustic noise [5]. In addition, to eliminate the effect of temperature variations, experiments were conducted at constant temperature in a thermally isolated chamber under ambient pressure.

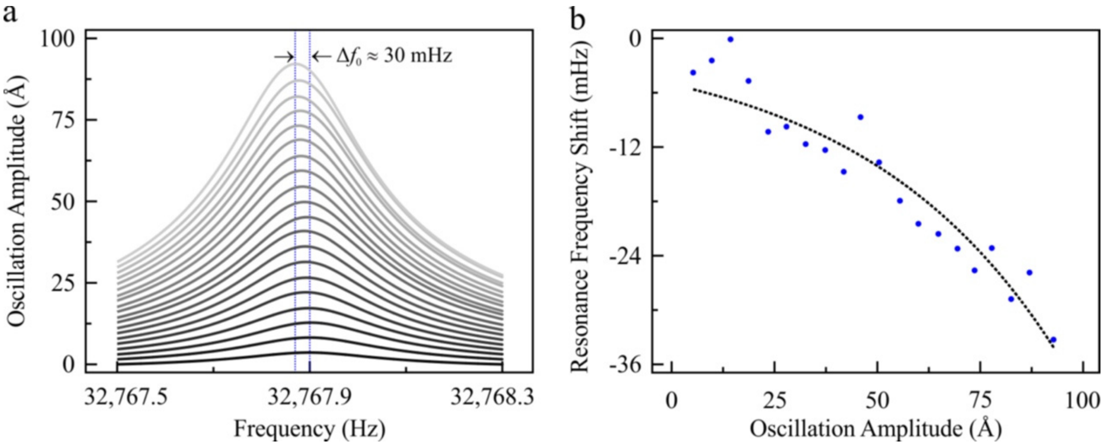

Figure S2: The measurement of the resonance frequency of an encapsulated tuning fork as a function of oscillation amplitude. (**a**) Frequency sweep experiments were conducted to determine the resonance frequency. The oscillation amplitude of the tuning fork (type-III, see Table S1 in part III of the supplemental materials for details of the tuning fork used for this specific experiment) is calibrated with the principle of energy dissipation [4]. (**b**) The resonance frequency decreases with increasing oscillation amplitude.

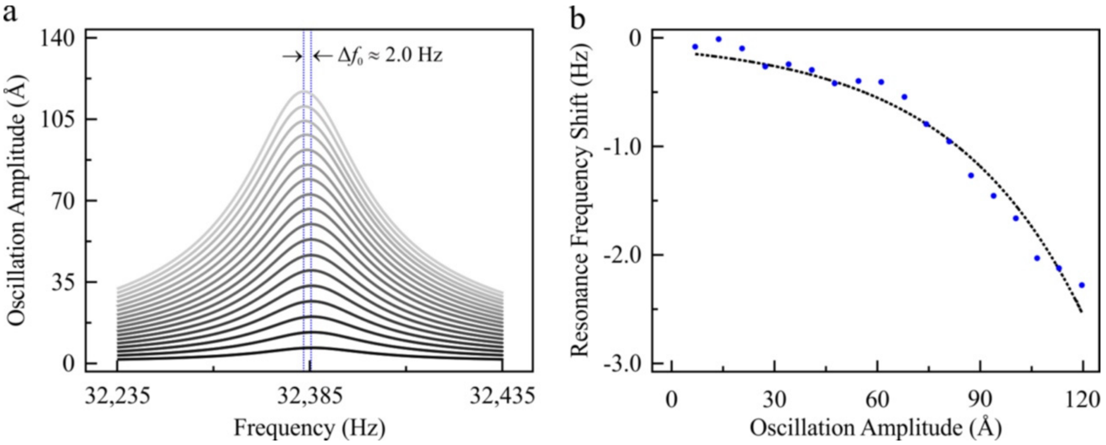

Figure S3: The measurement of the resonance frequency of a tuning fork in qPlus configuration. (**a**) Frequency sweep experiments were conducted to determine the resonance frequency. The oscillation amplitude of the qPlus sensor (based on tuning fork type-III, see Table S1 in part III of the supplemental materials for details of the tuning fork used for this specific experiment) is calibrated with the principle of energy dissipation [4]. (**b**) The resonance frequency changes up to 2 Hz when the oscillation amplitude is changed from 8 Å to 120 Å. The enhanced amplitude dependence is expected to be associated with the increased stress concentration at the clamped end of the prong due to the assembly process.

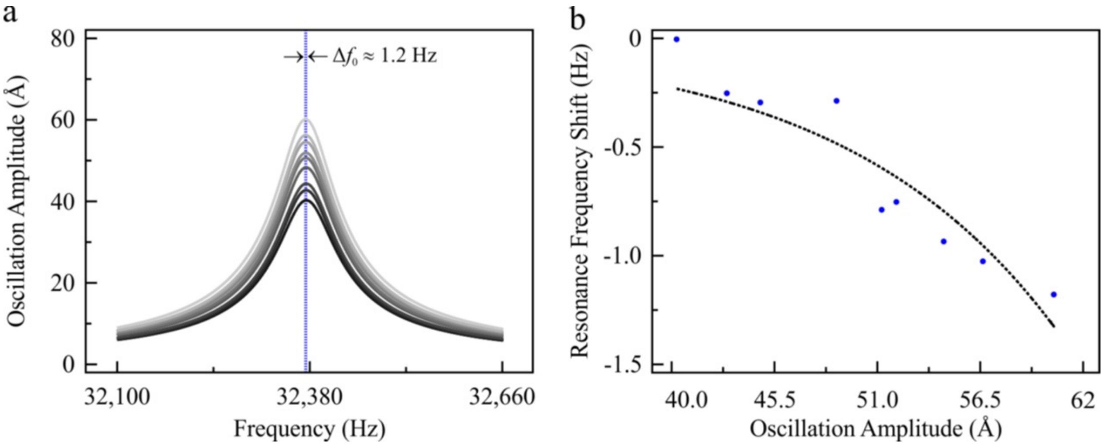

Figure S4. The measurement of the resonance frequency of a tuning fork in qPlus configuration. (**a**) Frequency sweep experiments are conducted to determine the resonance frequency. The oscillation amplitude of the qPlus sensor (based on tuning fork type-II, see Table S1 in part III of the supplemental materials for details of the tuning fork used for this specific experiment) is calibrated with the principle of energy dissipation [4]. (**b**) The resonance frequency changes up to 1.2 Hz when the oscillation amplitude is tuned up ~20 Å. The enhanced amplitude dependence is expected to be associated with the increased stress concentration at the clamped end of the prong due to the assembly process [6,7].

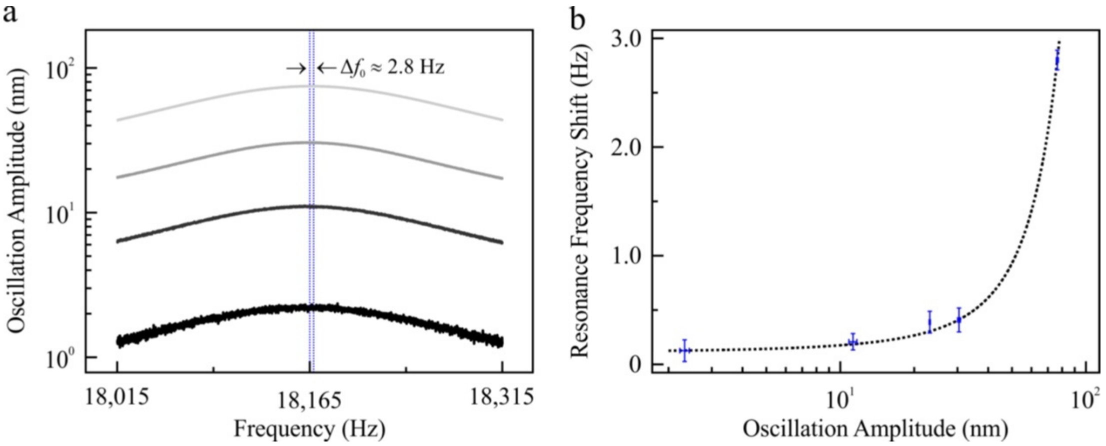

Figure S5: The measurement of the resonance frequency of a microfabricated silicon cantilever-I (OPUS 4XC-NN-A) as a function of oscillation amplitude. (**a**) Frequency sweep experiments were conducted to determine the resonance frequency. The frequency sweep experiments presented in this figure are averaged five times. A Lorentzian curve is fitted around the resonance amplitude to determine the resonance frequency. (**b**) The resonance frequency of the cantilever increases with the oscillation amplitude, which implies that geometric effects dominate [8]. Experiments were conducted under ambient conditions in a temperature-controlled room. To eliminate the effect of acoustic noise, experiments were performed in a quiet room under ambient pressure [5].

III. In-Plane Stress Near the Tuning-Fork Base

We briefly summarize the finite element method (FEM) approach to calculate the modal shape of the oscillation and the stress distribution of oscillating tuning fork-based sensors while further details for FEM calculations can be found elsewhere [3,9–12]. All calculations were performed using the COMSOL Multiphysics 4.4 structural mechanics software package (COMSOL Multiphysics, GmbH, Berlin, Germany).

The physical system is modeled when FEM calculations are conducted. Modeling the tuning fork for FEM calculations requires measuring the dimensions. We employed a calibrated optical microscope to obtain the dimensions of the tuning forks (Table S1). It is important to reflect the tuning fork's geometry accurately in regions where stress concentrations are expected, e.g., the region

between the prongs and where prongs are connected to the base of the tuning fork [3,12]. We did not include the gold coating, which has an average thickness of 200 Å, nor the notches at the tuning fork base to decrease the cost of computation and modeling. Neglecting these features in our FEM model has been justified as they are mainly important for electrical properties of tuning fork while having no substantial influence on the mechanical properties [3,11,13].

Table S1. Dimensions of tuning forks were measured with a calibrated light microscope.

	Width	Length of the Prong	Thickness
**Tuning Fork-I**	234 µm	2471 µm	90 µm
**Tuning Fork-II**	234 µm	2426 µm	131 µm
**Tuning Fork-III**	600 µm	3600 µm	250 µm

In addition to measuring geometric dimensions, assigning relevant material properties, such as Young's modulus, Poisson's ratio, mass density, and damping coefficient, is required. We used the material properties for quartz from the materials library of the FEM software with the mass density of a common epoxy glue (Epoxy Technology's EPO-TEK H72) as derived from the information provided in the manufacturer's data sheet and the damping coefficient of quartz and epoxy taken from References [14,15] (Table S2). Finally, we assumed that Macor does not contribute to the damping to speed up the calculations; doing so is justified due to the much smaller damping coefficient of Macor compared to quartz and, in particular, the epoxy, damping due to that Macor has virtually no influence on the simulation results [3,12].

Table S2. Material Properties Used for Finite Element Method (FEM) Calculations.

.	Young's Modulus (Gpa)	Poisson's Ratio	Mass Density (kg m^{-3})	Damping Coefficient
**Quartz**	82	0.17	2648	2×10^{-4}
**Epoxy**	7	0.35	1600	5×10^{-3}
**Macor**	300	0.222	3900	-

As Figure S6a summarizes, to determine the modal shape and the surface stress distribution due to the mechanical oscillation, boundary conditions are defined at the base of the Macor holder. Figure S6b shows that we increased the mesh density at the material interfaces and at regions where stress concentrations were expected. Eigenfrequency analysis of the assembly system reveals that the resonance frequency of the tuning fork assembly is 32,171 Hz and the tuning fork oscillates along the vertical direction. As Figure S6c discloses, that in-plane stress distribution was evident along the oscillation direction (with brighter colors presenting higher stress). It is clearly seen that stress is highest near the fixed end of the cantilevered free prong along the oscillation direction (in-plane) and can, in potential, induce variations of the resonance frequency [6–8,16].

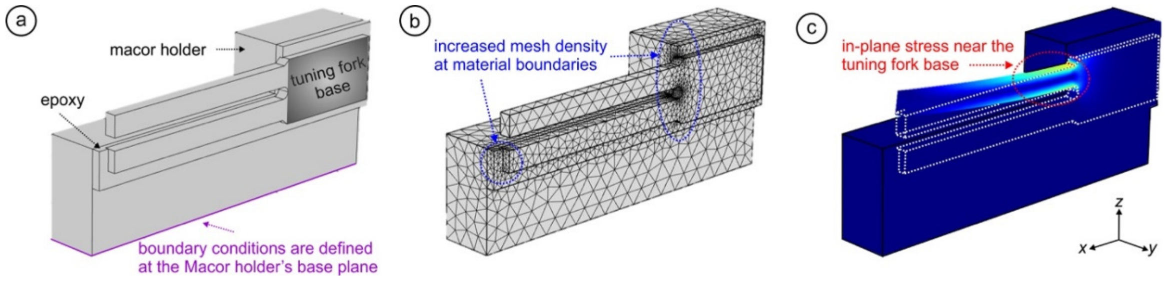

Figure S6: The assembled sensor setup and the modelling procedure to determine the modal shape and the surface stress distribution of tuning fork-based sensors. (**a**) Three-dimensional presentation of the sensor assembly including the tuning fork, Macor holder, and epoxy glue used to fix the tuning

fork to the base holder. (**b**) Enhanced mesh density is evident at the material boundaries and at the areas of stress concentrations. (**c**) The modal shape of the oscillation was calculated with eigenfrequency analysis. The first operational mode of the sensor assembly has a resonance frequency of 32,171 Hz and the oscillation is along *z*-direction. With brighter colors representing higher stress along the oscillation direction (in-plane), it is evident that the stress is the highest near the fixed end of the cantilevered free prong.

IV. Product of the Spring Constant with the Relative Resonance Frequency Shift

To investigate the convergence of the surface stress when the oscillation amplitude was increased from thermal limit to nanometer range, we calculated the stiffness of the tuning forks we used in our experiments. Experimental and theoretical approaches are available to calibrate the spring constant (k) of the probes used for scanning probe microscopy (SPM) experiments (see Reference [17–20] for detailed reviews). We used the FEM-based technique to calculate the spring constant of tuning forks (*see section III of the SM for details*). As Figure S7a summarizes, while keeping one of the prongs and the base of the tuning fork rigid, we applied force along *z*-direction to the end of the free prong. We swept the force from 1 µN to 100 µN with 1 µN steps and measured the displacement, Δz. The next step was fitting a first-order polynomial to find the spring constant of the tuning fork by using Hooke's law.

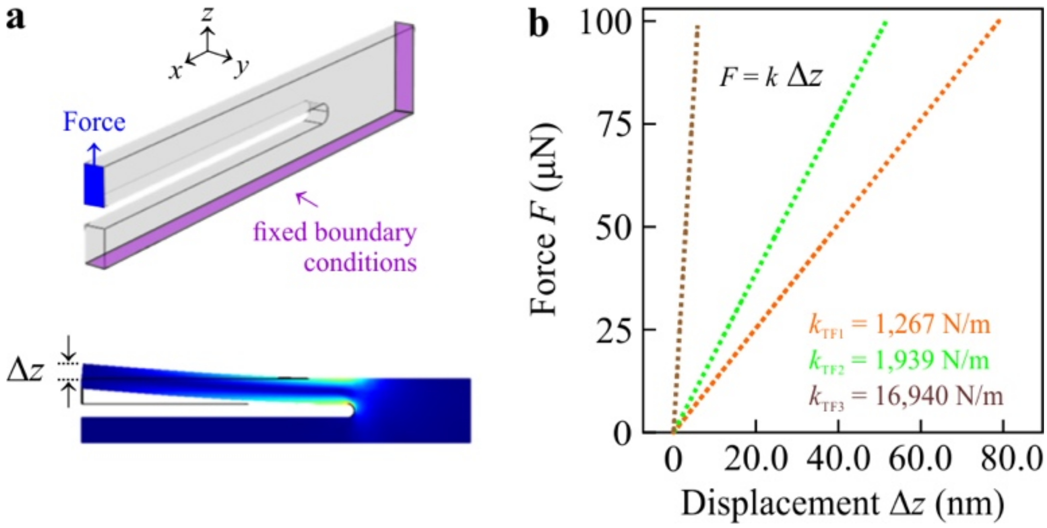

Figure S7: Calibration of spring constant with finite element methods. (**a**) One of the prongs and the base of the tuning fork were fixed, while force was applied to the end of the free prong along the *z*-direction. The exerted force deforms the prong and the displacement at the end of the prong, Δz, was measured. (**b**) The slope of the force versus displacement curve is equal to the spring constant of the tuning fork, k.

Summarized in Figure S7b, we found consistent spring constant values with earlier experimental and computational results for similar tuning forks [1,9–11,21]. As outlined by different groups, calibration of spring constant with FEM techniques deviated up to 5% with respect to dynamic experimental results [3,9,12,22].

As Figure 1 in the main text shows, tuning forks were excited with very similar oscillation amplitudes, i.e., all in the nanometer range. For this reason, the total surface stress is assumed to be in the same range. The multiplication of the relative resonance frequency shift (Figure 1 in the main text) with the equivalent spring constant of tuning forks ($k_{eff} = 2k$, where k is the stiffness of a single prong), calculated with the finite element method, gives a constant product of 3,460 ± 160 (kg/s^3). Such a convergence implies that the in-plane surface stress is the governing physical phenomena that leads the amplitude dependent resonance frequency. In addition, we want to underline that even if

the amplitude dependence of the resonance frequency has a physical origin other than the surface stress effect (for tuning forks) or geometric effect (for cantilevers), consequences on the spectroscopy experiments will always exist (*please see the main text for a detailed discussion*).

V. Computational Methods Employed in the Numerical Analysis of Resonance Shifts

Following a commonly used approach for dynamic atomic force microscopy simulations, we solved the equation of motion of a damped harmonic oscillator with external excitation and non-linear tip-sample interaction force [23–27], as follows:

$$m\ddot{z}(t) + \frac{2\pi f_0 m}{Q}\dot{z}(t) + c_z[z(t) - d] = a_{\mathrm{d}} c_z \cos(2\pi f_{\mathrm{d}} t) + F_{\mathrm{ts}}[z(t), \dot{z}(t)], \quad \text{(S1)}$$

where $z(t)$ is the position of the tip as a function of time t (with $z = d$ denoting the distance of the tip relative to the sample when the cantilever is undeflected); m, f_0, Q, and c_z are the effective mass, the first eigenfrequency, the quality factor, and the spring constant of the oscillator, respectively. In Equation (S1), the terms on the left reflect the standard terms for a damped harmonic oscillator, while the first term on the right represents the external excitation of the oscillator with excitation amplitude a_d and excitation frequency f_d. The second term on the right side finally symbolizes the non-linear tip sample interaction force F_{ts}, which may depend both on the tip's time-dependent position z as well as its instantaneous velocity $\dot{z}$. Neglecting a possible velocity dependence, we chose F_{ts} in agreement with previous literature [25–27] as a combination of a van der Waals-type sphere-over-flat interaction [28] for the attractive regime ($z \geq z_0$) and a contact force ($z < z_0$) that follows Maugis' approximation to the Derjaguin–Muller–Toporov model (DMT-M) [29–31], which is often referred to as the Hertz-plus-offset model [32], as follows:

$$F_{\mathrm{ts}}(z) = \begin{cases} -\dfrac{A_{\mathrm{H}} R}{6z^2} & \text{for } z \geq z_0 \\ \dfrac{4}{3} E^* \sqrt{R}\left(z_0 - z\right)^{3/2} - \dfrac{A_{\mathrm{H}} R}{6z_0^2} & \text{for } z < z_0 \end{cases} \quad \text{(S2)}$$

where A_{H} = 0.2 aJ is the Hamaker constant, R = 10 nm the radius of the tip's apex, z_0 = 0.3 nm the distance at which the contact is established, and $E^* = ((1-\nu_t^2)/E_t + (1-\nu_s^2)/E_s)^{-1}$ the combined elastic modulus of the tip and sample (with E_t = 130 GPa as the Young's modulus of the tip, E_s = 1 GPa as the Young's modulus of the sample, and $\nu_t = \nu_s = 0.3$ as the Poisson ratios of tip and sample, respectively). To describe the oscillator, we chose c_z = 2,000 N/m, $f_d = f_0$ = 22,000 Hz, and Q = 10,000; these values reflect typical parameters for a tuning fork glued on a holder in qPlus configuration, which represents the current most common oscillator choice for high-resolution vacuum-based atomic force microscopy.

Equation (S1) was then solved by employing a previously derived analytical solution for the tip-sample motion, which is, however, defined for conservative tip-sample interactions only [26]. Finally, Equation (S3) details the numerical integration method we used for reconstructing the tip-sample interaction potential, U_{ts}, from data obtained with FM-type force spectroscopy introduced by Sader and Jarvis, which represents the most widely used reconstruction protocol for this case [33]. It results in the following equation:

$$U_{\mathrm{ts}}(D) = 2c_z \int_D^{\infty} \frac{f_0 - f_{\mathrm{res}}}{f_0} \left[(z - D) + \sqrt{\frac{A}{16\pi}}\sqrt{z - D} + \frac{A^{3/2}}{\sqrt{2(z - D)}} \right] dz \quad \text{(S3)}$$

Note that U_{ts} is given as a function of nearest tip-sample distance D, which distinguishes itself from the distance d the tip has to the surface when the cantilever is undeflected by $D = d - A$, where $f_{res}(D)$ represents the cantilever's distance-dependent resonance frequency (i.e., $\Delta f = f_0 - f_{res}$). With the knowledge of $U_{ts}(D)$, the tip-sample force, F_{ts}, as a function of D can easily be recovered for both cases by calculating its negative gradient, ($F_{ts}(D) = -\partial U_{ts}/\partial D$).

In passing, we want to mention that the error induced by the amplitude dependence of the resonance frequency is independent of the (model) tip-sample interaction used in our numerical analysis. According to Equation (3) of the main text, the measured frequency shift is presented by the following:

$$\Delta f_{measured} = \Delta f_{tip-sample} + \Delta f_{error} \tag{S4}$$

In equation (S4), $\Delta f_{measured}$ is the total resonance frequency shift which is equal to the summation of $\Delta f_{tip-sample}$, the frequency shift due to the tip-sample interaction, and Δf_{error}, the frequency shift induced by the amplitude dependence of the resonance frequency. The value $\Delta f_{tip-sample}$ is calculated using the model tip-sample interaction force and Δf_{error} is calculated using the experimental dependence of the resonance frequency on amplitude for different amplitude errors assumed (*see the main text for details and/or section VI of the supplemental materials for details*). The error in the reconstructed tip-sample interaction potential is calculated by the following:

$$U_{\mathrm{error}}(D) = U_{\mathrm{measured}}(D) - U_{\mathrm{ts}}(D). \tag{S5}$$

By inserting Equation (S4) into the Equation (S3) and rearranging Equation (S5), the error in the tip-sample interaction potential can be expressed by the following:

$$\begin{aligned} U_{\mathrm{error}}(D) = c_z \int_D^{\infty} \frac{\Delta f_{tip-sample} + \Delta f_{error}}{f_0} \left[(z - D) + \sqrt{\frac{A}{16\pi}} \sqrt{z - D} + \frac{A^{3/2}}{\sqrt{2(z - D)}} \right] dz \\ - c_z \int_D^{\infty} \frac{\Delta f_{tip-sample}}{f_0} \left[(z - D) + \sqrt{\frac{A}{16\pi}} \sqrt{z - D} + \frac{A^{3/2}}{\sqrt{2(z - D)}} \right] dz \end{aligned} \tag{S6}$$

With the rearrangement of Equation (S6), we have the following expression:

$$U_{\mathrm{error}}(D) = c_z \int_D^{\infty} \frac{\Delta f_{error}}{f_0} \left[(z - D) + \sqrt{\frac{A}{16\pi}} \sqrt{z - D} + \frac{A^{3/2}}{\sqrt{2(z - D)}} \right] dz \tag{S7}$$

As Equation (S7) reveals, the error in the reconstructed tip-sample interaction potential (or force, $F_{\mathrm{error}}(D) = -\partial U_{\mathrm{error}}/\partial D$), is independent of the tip-sample interaction model used in our calculations. The error due to the amplitude dependence of the resonance frequency is due to intrinsic properties of the oscillating probe used for the experiments. In the following, we employed a model tip-sample interaction law to better explain the algorithm to correct the resonance frequency shift data in order to eliminate the effect of the amplitude dependence of the resonance frequency (see part VI of the supplemental materials for details).

VI. Algorithm to Correct the Resonance Frequency Shift Data to Eliminate the Effect of Amplitude Dependence of Resonance Frequency

Figure S8 summarizes the algorithm to correct the measured resonance frequency shift to eliminate the effect of the amplitude dependence of the resonance frequency. This effect should be particularly corrected when there is a sudden change in the tip-sample interaction. However, for experiments that are conduced slow enough, the error in the oscillation amplitude will oscillate around the set point and the effect of the resonance dependence of the oscillation amplitude cancels out. The correction procedure for the amplitude dependence of the resonance frequency should be applied to each sensor when they are introduced to the microscope. After the introduction of the sensor to the microscope, the first step is to calibrate the oscillation amplitude to find the conversion factor between the electrical readout and the mechanical oscillation amplitude. In our experiments, we used the principle of energy dissipation to calibrate the oscillation amplitude of tuning forks and qPlus sensors [4], while details of alternative oscillation amplitude calibration techniques can be found elsewhere [9]. We calibrated the oscillation amplitude of the cantilevers using the thermal

excitation technique, which was performed in a quiet room to eliminate the effect of acoustic noise [5]. With the calibration of the oscillation amplitude, frequency sweep experiments at different resonance amplitudes are conducted (*see section II of the supplemental materials for experimental details of frequency sweep experiments*). The next phase is to fit the resonance frequency shift as a function of the oscillation amplitude, $\Delta f(A)$, where Δf is the variation of the resonance frequency and A is the oscillation amplitude (*see Figure 2 of the main text for an example*). With the measurement of the oscillation amplitude dependence of the resonance frequency, the correction of the amplitude dependence of the resonance frequency can be applied to measured frequency shifts. Towards this end, the oscillation amplitude should be recorded during the experiments and the oscillation amplitude error (A_{error}) should be used to deconvolve the resonance frequency shift due to amplitude variation. As a final step, the frequency shift due to amplitude error (Δf_{error}) is subtracted from the measured frequency shift ($\Delta f_{measured}$) and the frequency shift due to tip-sample interaction ($\Delta f_{tip-sample}$) is obtained. Although we detailed the algorithm to correct the resonance frequency shift data to eliminate the effect of the amplitude dependence of the resonance frequency for force spectroscopy experiments, a similar procedure can be applied to experiments where the accuracy resonance frequency shift information is important, e.g., Kelvin probe force microscopy, pump-probe atomic force microscopy, and infra-red atomic force microscopy (*see the main text for a discussion*).

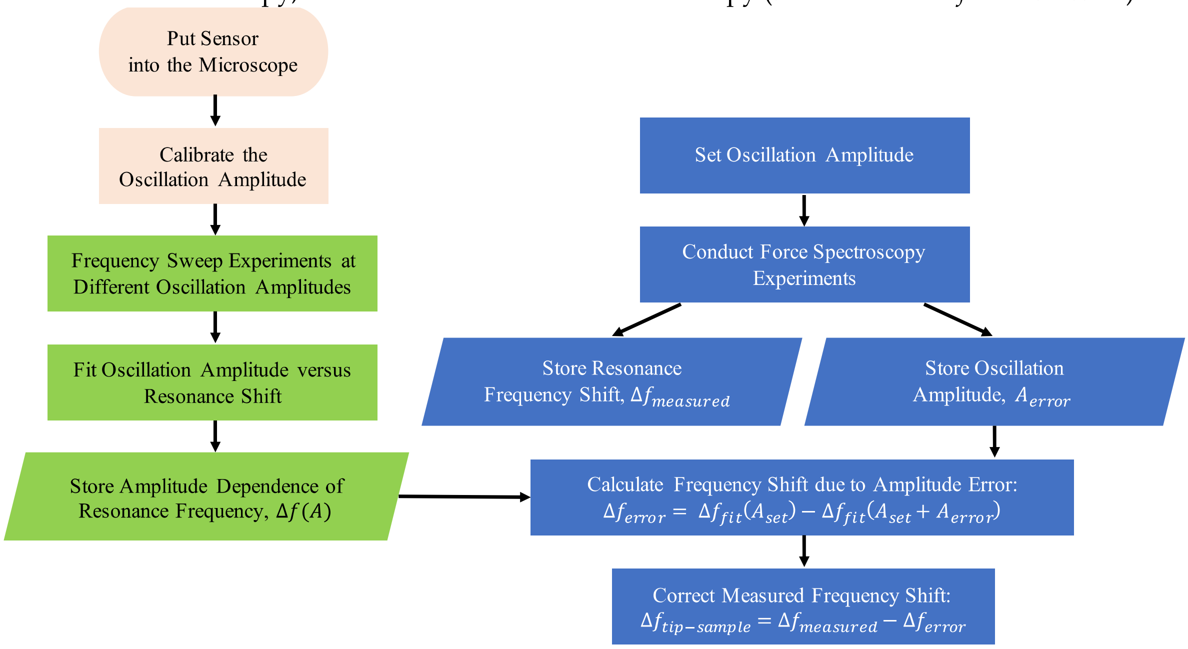

Figure S8: Summary of the algorithm to correct the resonance frequency shift data to eliminate the effect of the amplitude dependence of the resonance frequency**.** This algorithm provides a general pathway to correct the effect of the amplitude dependence of the resonance frequency. If the experiment is conducted slow enough to control the oscillation amplitude around the amplitude set point, the effect of amplitude dependent resonance frequency shift may cancel out without the need of the correction. However, if there is a sudden change in the tip-sample interaction due to imaging speed or the nature of the experiment, this proposed algorithm can be implemented to enhance the accuracy of the experiment.

References

1. Giessibl, F.J. High-speed force sensor for force microscopy and profilometry utilizing a quartz tuning fork. *Appl. Phys. Lett.* **1998,** *73*, 3956–3958.
2. Karrai, K.; Grober, R.D. Piezoelectric tip-sample distance control for near field optical microscopes. *Appl. Phys. Lett.* **1995,** *66*, 1842–1844.
3. Dagdeviren, O.E.; Schwarz, U.D. Numerical performance analysis of quartz tuning fork-based force sensors. *Meas. Sci. Technol.* **2017,** *28*, 015102.
4. Dagdeviren, O.E.; Miyahara, Y.; Mascaro, A.; Grütter, P. Calibration of the oscillation amplitude of electrically excited scanning probe microscopy sensors. *Rev. Sci. Instrum.* **2019,** *90*, 013703.

5. Mascaro, A.; Miyahara, Y.; Dagdeviren, O.E.; Grütter, P. Eliminating the effect of acoustic noise on cantilever spring constant calibration. *Appl. Phys. Lett.* **2018,** *113*, 233105.
6. Lachut, M.J.; Sader, J.E. Effect of Surface Stress on the Stiffness of Cantilever Plates. *Phys. Rev. Lett.* **2007,** *99*, 206102.
7. Lachut, M.J.; Sader, J.E. Effect of surface stress on the stiffness of thin elastic plates and beams. *Phys. Rev. B: Condens. Matter* **2012,** (8), 085440.
8. Karabalin, R.B.; Villanueva, L.G.; Matheny, M.H.; Sader, J.E.; Roukes, M.L. Stress-Induced Variations in the Stiffness of Micro- and Nanocantilever Beams. *Phys. Rev. Lett.* **2012,** *108*, 236101.
9. Georg Hermann, S.; Markus, H.; Hans-Peter, R. Recipes for cantilever parameter determination in dynamic force spectroscopy: spring constant and amplitude. *Nanotechnology* **2007,** *18*, 255503.
10. van Vörden, D.; Lange, M.; Schmuck, M.; Schmidt, N.; Möller, R. Spring constant of a tuning-fork sensor for dynamic force microscopy. *Beilstein J. Nanotechnol.* **2012,** *3*, 809–816.
11. Falter, J.; Stiefermann, M.; Langewisch, G.; Schurig, P.; Hölscher, H.; Fuchs, H.; Schirmeisen, A. Calibration of quartz tuning fork spring constants for non-contact atomic force microscopy: direct mechanical measurements and simulations. *Beilstein J. Nanotechnol.* **2014,** *5*, 507–516.
12. Dagdeviren, O.E.; Schwarz, U.D. Optimizing qPlus sensor assembly for simultaneous scanning tunneling and noncontact atomic force microscopy operation based on finite element method analysis. *Beilstein J. Nanotechnol.***2017,** *8*, 657–666.
13. Whang Y.J.; Chris, T. G.-C. A Study of Small Sized Quartz Tuning Fork Using Finite Element Analysis. Available online: http://www.te.chiba-u.jp/~ken/Symp/Ishou2008/proceeding/10.%20A%20Study%20of%20Small%20Sized%20Quartz%20Tuning%20Fork%20Using%20Finite%20Element%20Analysis.pdf (accessed on 15 September 2019)
14. Malatkar, P. Nonlinear Vibrations of Cantilever Beams and Plates. Available online: https://vtechworks.lib.vt.edu/handle/10919/28301 (accessed on 15 September 2019)
15. Petyt, M. *Introduction to Finite Element Vibration Analysis*. 2nd ed.; Cambridge University Press: Cambridge, 2010.
16. Ruz, J.J.; Pini, V.; Malvar, O.; Kosaka, P.M.; Calleja, M.; Tamayo, J. Effect of surface stress induced curvature on the eigenfrequencies of microcantilever plates. *AIP Adv.* **2018,** *8*, 105213.
17. Burnham, N.A.; Chen, X.; Hodges, C.S.; Matei, G.A.; Thoreson, E.J.; Roberts, C.J.; Davies, M.C.; Tendler, S.J.B. Comparison of calibration methods for atomic-force microscopy cantilevers. *Nanotechnology* **2003,** *14*, 1.
18. Butt, H.-J.; Cappella, B.; Kappl, M. Force measurements with the atomic force microscope: Technique, interpretation and applications. *Surf. Sci. Rep.* **2005,** *59*, 1–152.
19. Cook, S.M.; Schäffer, T.E.; Chynoweth, K.M.; Wigton, M.; Simmonds, R.W.; Lang, K.M. Practical implementation of dynamic methods for measuring atomic force microscope cantilever spring constants. *Nanotechnology* **2006,** *17*, 2135.
20. Palacio, M.L.B.; Bhushan, B. Normal and Lateral Force Calibration Techniques for AFM Cantilevers. *Crit. Rev. Solid State Mater. Sci.* **2010,** *35*, 73–104.
21. Albers, B.J.; Liebmann, M.; Schwendemann, T.C.; Baykara, M.Z.; Heyde, M.; Salmeron, M.; Altman, E.I.; Schwarz, U.D. Combined low-temperature scanning tunneling/atomic force microscope for atomic resolution imaging and site-specific force spectroscopy. *Rev. Sci. Instrum.* **2008,** *79*, 033704.
22. Chen, B.-Y.; Yeh, M.-K.; Tai, N.-H. Accuracy of the Spring Constant of Atomic Force Microscopy Cantilevers by Finite Element Method. *Anal. Chem.* **2007,** *79*, 1333–1338.
23. Anczykowski, B.; Krüger, D.; Babcock, K.L.; Fuchs, H. Basic properties of dynamic force spectroscopy with the scanning force microscope in experiment and simulation. *Ultramicroscopy* **1996,** *66*, 251–259.
24. Dagdeviren, O.E.; Götzen, J.; Hölscher, H.; Altman, E.I.; Schwarz, U.D. Robust high-resolution imaging and quantitative force measurement with tuned-oscillator atomic force microscopy. *Nanotechnology* **2016,** *27*, 065703.
25. Hölscher, H. Theory of phase-modulation atomic force microscopy with constant-oscillation amplitude. *J. Appl. Phys.* **2008,** *103*, 064317.
26. Hölscher, H.; Schwarz, U.D., Theory of amplitude modulation atomic force microscopy with and without Q-Control. *Int. J. Non Linear Mech.* **2007,** *42*, 608–625.
27. Dagdeviren, O.E.; Zhou, C.; Altman, E.I.; Schwarz, U.D., Quantifying Tip-Sample Interactions in Vacuum Using Cantilever-Based Sensors: An Analysis. *Phys. Rev. Appl.* **2018,** *9*, 044040.
28. Israelachvili, J. *Intermolecular and Surface Forces*. 2nd ed.; Academic Press: London, UK, 1991.
29. Schwarz, U.D. A generalized analytical model for the elastic deformation of an adhesive contact between a sphere and a flat surface. *J. Colloid Interface Sci.* **2003,** *261*, 99–106.

30. Maugis, D.; Gauthier-Manuel, B. JKR-DMT transition in the presence of a liquid meniscus. *J. Adhes. Sci. Technol.* **1994,** *8*, 1311–1322.
31. Greenwood, J.A.; Johnson, K.L. An alternative to the Maugis model of adhesion between elastic spheres. *J. Phys. D: Appl. Phys.* **1998,** *31*, 3279.
32. Schwarz, U.D.; Zwörner, O.; Köster, P.; Wiesendanger, R. Quantitative analysis of the frictional properties of solid materials at low loads. I. Carbon compounds. *Phys. Rev. B: Condens. Matter* **1997,** *56*, 6987–6996.
33. Sader, J.E.; Jarvis, S.P. Accurate formulas for interaction force and energy in frequency modulation force spectroscopy. *Appl. Phys. Lett.* **2004,** *84*, 1801–1803.
